# Diagnosis of *Plasmodium vivax* by Loop-Mediated Isothermal Amplification in Febrile Patient Samples from Loreto, Perú

**DOI:** 10.4269/ajtmh.20-0212

**Published:** 2020-08-03

**Authors:** Oscar Nolasco, Beronica Infante, Juan Contreras-Mancilla, Sandra Incardona, Xavier C. Ding, Dionicia Gamboa, Katherine Torres

**Affiliations:** 1 Instituto de Medicina Tropical “Alexander von Humboldt” Universidad Peruana Cayetano Heredia, Lima, Perú;; 2 Laboratorios de Investigación y Desarrollo, Facultad de Ciencias y Filosofía, Universidad Peruana Cayetano Heredia, Lima, Perú;; 3 Foundation for Innovative New Diagnostics, Geneva, Switzerland;; 4 Departamento de Ciencias Celulares y Moleculares, Facultad de Ciencias y Filosofía, Universidad Peruana Cayetano Heredia, Lima, Perú

## Abstract

*Plasmodium vivax* is co-endemic with *Plasmodium falciparum* in Peru*,* and optimum management requires distinguishing these two species in the blood of patients. For the differential identification of *P. vivax* and other *Plasmodium* spp., the Loopamp^TM^ Malaria Pan Detection Kit in combination with the Loopamp Malaria Pv Detection Kit (Eiken Chemical Co. Ltd., Tokyo, Japan) was used to evaluate 559 whole blood samples collected in 2017 from febrile patients with suspected malaria attending different health facilities in the Loreto region. The Loopamp Malaria Pan Detection Kit showed a sensitivity of 87.7% (95% CI: 83.5–91.9) and a specificity of 94.4% (95% CI: 91.9–96.9) and good agreement with PCR (Cohen’s kappa 0.8266, 95% CI: 0.7792–0.874). By comparison, the Loopamp Malaria Pv Detection Kit showed a similar sensitivity (84.4%, 95% CI: 79.0–89.7) and specificity (92.4%, 95% CI: 89.7–95.0) and substantial agreement with PCR (Cohen’s kappa: 0.7661, 95% CI: 0.7088–0.8234).

In the Peruvian Amazonia, good sensitivity and specificity for diagnosis of *Plasmodium* infection are essential for efficient malaria control. In this region, there are two species of *Plasmodium* with different prevalence; the reported incidence of *Plasmodium vivax* infectious is nearly four times higher than *Plasmodium falciparum* infections.^1^ The detection and identification are a challenge because of the high prevalence of submicroscopic and asymptomatic infections confirmed by epidemiological studies.^2,3^ Specific detection of *P. vivax* is required so that a radical cure combining chloroquine and primaquine to eliminate both blood- and liver-stage parasites can be administered.^4^

Microscopy including thin and thick blood smear is the most commonly used diagnostic method for detection and identification of *Plasmodium* and as such remains a standard of practice. Although microscopy can achieve an excellent detection limit when overseen by an expert (5–20 parasites per microliter of blood [p/µL]), this method is often associated with misdiagnosis at points of care (POC) when performed by a health worker with limited proficiency and in infections with low parasitemia (10–100 p/µL).^5,6^ Rapid diagnostic tests (RDTs) represent an alternative for malaria diagnosis by offering an easy-to-use and quick method. Their sensitivity for *P. falciparum* detection is approximately 100 p/µL; however, current RDTs for *P. vivax* are usually slightly less sensitive, translating to a significant proportion of false negatives because of the low parasitemia typically associated with infections by this species.^7–9^ Molecular diagnostic methods can achieve a sensitivity as low as 0.02 p/µL^10^ and are understandably the best option to detect and identify all *Plasmodium* infections, including those in asymptomatic patients.^11^ However, only isothermal amplification techniques such as the loop-mediated isothermal amplification (LAMP) can be effectively applied in areas with limited laboratory capacity because DNA amplification occurs at a constant temperature and the amplification product can be visually detected by turbidity or fluorescence, without any additional sample manipulation.^12^

The commercially available Loopamp Pan/Pf Malaria Kit has been previously used in multiple settings, including in the Loreto region in Peruvian Amazonia, showing a high operational capacity in the field and a high sensitivity.^13^ Herein, we report the clinical evaluation of a *P. vivax* infection by using the Loopamp™ Malaria Pv Detection Kit (Human, ref. 975000), which complements the already available LAMP products designed for detection of pan *Plasmodium* spp. and *P. falciparum*.

The Loopamp™ Malaria Pan Detection Kit (Human, ref. 977000) and the *P. vivax* commercial assay were evaluated in a retrospective laboratory study against 559 frozen ethylenediaminetetraacetic acid-anticoagulated venous whole blood samples. Samples were collected from febrile patients consecutively attending four different health facilities located in the communities of San Juan, Lupuna, Santa Clara, and Quistococha in the Loreto region from March to June 2017. The study was approved by the Institutional Ethical Committee of Universidad Peruana Cayetano Heredia (code 100068).

All the samples were evaluated by two independent microscopy readings and by molecular diagnostic methods in two reference laboratories. A real-time PCR diagnosis with a species-specific melting probe and unidirectional sequencing by using the noncoding region between the *cytb* and *cox1* genes was performed at Labor Limbach Laboratory (Heidelberg, Germany) for species determination.^14^ A second analysis by nested PCR and bidirectional sequencing for species determination were performed at Microsynth laboratory (Balgach, Switzerland), using the *cytb* gene of the *Plasmodium* mitochondrial DNA.^15^

The LAMP kits were used according to the manufacturer’s instructions.^16^ In brief, nucleic acid was extracted using the boil and spin method, where 60 μL of whole blood sample was dispensed into a 1.5-mL tube containing 60 μL of DNA extraction buffer (400 mM NaCl, 40 mM Tris pH 6.5% and 0.4% SDS), mixed for 5 minutes at 95°C in a heat block, and centrifuged at 10,000 g for 3 minutes. The supernatant was recovered, and 30 μL was transferred into a tube containing 345 μL of sterile water. The extracted and diluted DNA (30 μL) was added to the Pan LAMP and Pv LAMP reaction tubes, with resuspension of the reagents according to the manufacturer’s instructions. Samples were incubated for 40 minutes at 65°C in a heat block, followed by 5 minutes at 80°C to stop the reaction. DNA amplification was detected based on fluorescence observed in the reaction mix when exposed to an ultraviolet lamp. Each LAMP reaction was judged three times, and the final report was the result of two or three equal visual observations.

Of the 559 samples, 225 (40.6%) showed positive reaction to *Plasmodium* by the Loopamp Malaria Pan Detection Kit, whereas 180 (32.2%) samples showed positive reaction to *P. vivax* by the Loopamp Malaria Pv Detection Kit. PCR (composite of Limbach and Microsynth laboratory results) detected 236 (42.2%) samples as *Plasmodium* infections; of these, 173 (73.3%) were identified as *P. vivax*, 57 (24.2%) as *P. falciparum*, and six (2.5%) as mixed-species infections. Neither *Plasmodium ovale* nor *Plasmodium malariae* infections were identified. By microscopy, a total of 182 (32.6%) samples were identified as *Plasmodium* infections; of these, 138 (75.8%) were identified as *P. vivax*, 42 (23.8%) as *P. falciparum*, and two (1.1%) as mixed-species infections.

A good agreement to detect *Plasmodium* infections was shown between the Loopamp Malaria Pan Detection Kit and the combined PCR techniques (Cohen’s Kappa 0.8266, 95% CI: 0.7792–0.874). Using the combined PCR as the reference method, the Loopamp Malaria Pan Detection Kit showed a sensitivity of 87.7% (95% CI: 83.5–91.9) and a specificity of 94.4% (95% CI: 91.9–96.9). At the same time, microscopy showed a lower sensitivity of 77.1% (95% CI: 71.8–82.5) and a higher specificity of 100% (Table 1).

**Table 1:** Performance evaluation of microscopy and Loopamp Malaria Pan Detection Kit for *Plasmodium* detection, using PCR as the reference method

	Statistic	Microscopy		Pan LAMP	
*Plasmodium* infections	True negative	323		305	
	True positive	182		207	
	False positive	0		18	
	False negative	54		29	
	Sensitivity (95% CI)	77.1	(71.8–82.5)	87.7	(83.5–91.9)
	Specificity (95% CI)	100		94.4	(91.9–96.9)
	Positive predictive value, (95% CI)	100		92	(88.5–95.5)
	Negative predictive value (95% CI)	85.7	(82.1–89.2)	91.3	(88.3–94.3)
	Cohen’s kappa	0.7957 SE 0.0259	(0.745–08464)	0.8266 SE 0.0242	(0.7792–0.874)

SE = standard error.

For detection of *P. vivax* infections, the Loopamp Malaria Pv Detection Kit showed a sensitivity of 84.4% (95% CI: 79.0–89.7) and a specificity of 92.4% (95% CI: 89.7–95.0), with a substantial agreement with PCR (Cohen’s kappa: 0.7661, 95% CI: 0.7088–0.8234). (Table 2), whereas the microscopy had a sensitivity of 77.7% (95% CI: 71.6–83.8) and a specificity of 99.7% (95% CI: 99.2–100), with Cohen’s kappa of 0.8212 (95% CI: 0.7693–0.8731). Considering a scenario where only Pan LAMP-positive samples would be tested with the Loopamp Malaria Pv Detection Kit, the positive predictive value and negative predictive value would have been 83.9 (78.5–89.3) and 92.6 (90–95.2), respectively.

**Table 2:** Performance evaluation of microscopy and the Loopamp Malaria Pv Detection Kit for *Plasmodium vivax* identification, using PCR as the reference method

	Statistic	Microscopy		Pv loop-mediated isothermal amplification	
*Plasmodium vivax infections*	True negative	379		351	
	True positive	139		151	
	False positive	1		29	
	False negative	40		28	
	Sensitivity (95% CI)	77.7	(71.6–83.8)	84.4	(79.0–89.7)
	Specificity (95% CI)	99.7	(99.2–100)	92.4	(89.7–95.0)
	Positive predictive value (95% CI)	99.3	(99.7–100)	83.9	(78.5–89.3)
	negative predictive value (95% CI)	90.5	(87.6–93.3)	92.6	(90.0–95.2)
	Cohen’s kappa	0.8212 SE 0.0265	(0.7693–0.8731)	0.7661 SE 0.0292	(0.7088–0.8234)

LAMP = loop-mediated isothermal amplification; SE = standard error.

The Loopamp Malaria Pv Detection Kit identified 29 (5.1%) positive samples that were not detected as *P. vivax* by PCR techniques (Figure 1). One of these was a sample classified as a mixed infection by microscopy and as a *P. falciparum* infection by PCR; three were identified as *P. falciparum* only by PCR; 10 were identified as *P falciparum* by microscopy and PCR, suggesting a failure in the specificity of the primer set; and the remaining 15 apparently false-positive sample were detected as *Plasmodium* by the Loopamp Malaria Pan Detection Kit. This finding of apparent false positives was reported in previous studies of the Loopamp Malaria Pan Detection Kit and could be explained by discrepancies related to the stochastic processes expected in detection of low parasitemia infections.^13,17–19^

**Figure 1.**
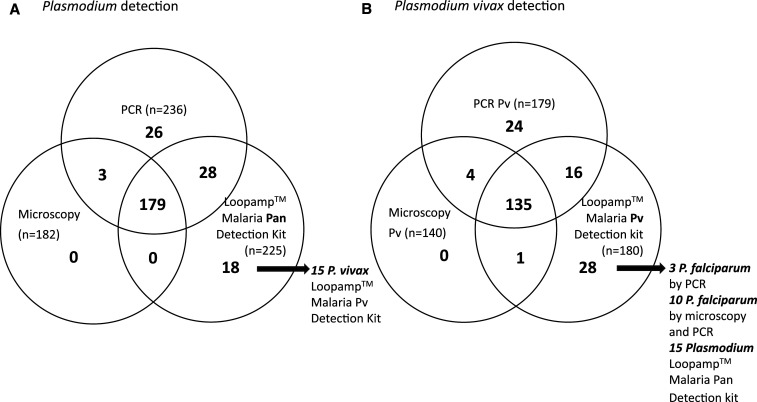
*Plasmodium* (**A**) *Plasmodium vivax* (**B**) infection identification according to three diagnostic methods.

The low density may have influenced the diagnostic accuracy; for seven of the 15 apparently false-positive samples, interobserver discrepancy was noted when reading the results of both LAMP assays. The interobserver differences in the Loopamp Malaria Pv Detection Kit was 9.1% (51/559), whereas for the Loopamp Malaria Pan Detection Kit was 7.5% (42/559).

In conclusion, the performance of a new commercial Loopamp Malaria Pv Detection Kit was consistent with that of an already available Loopamp Malaria Pan Detection Kit, with a superior sensitivity than microscopy performed in this study by two independent readers and highly skilled personnel. This suggests that the LAMP diagnosis could be very useful in rural and remote areas of Peru, where malaria incidence is high, and microscopic diagnosis does not always meet the quality requirements owing to limited resources. However, more studies would be needed to evaluate the operational feasibility and cost-effectiveness of implementing LAMP in such settings than the costs and operational difficulties of maintaining high-quality microscopy diagnosis.

The definite identification of *P. vivax*, which is needed to provide adequate radical treatment, could be effectively performed by combining the use of a Loopamp Malaria Pan Detection Kit and Loopamp Malaria Pv Detection Kit. However, because of the rate of false identification of *P. falciparum* as *P. vivax* seen in this study (7.2% [13/180]), the combined use with the Loopamp™ Malaria Pan/Pf Detection Kit would be a much better option for making adequate treatment decisions.

This report focuses on the use of LAMP with febrile individuals attending a health facility, but the performance of this new assay should also be evaluated in asymptomatic low parasitemia infections, where the superior sensitivity of LAMP would likely be more critical. There might be various clinical scenarios where LAMP will likely be a useful tool in malaria elimination programs; however, important aspects for its implementation, such as training of health workers and management of shelf life and storage conditions of the reagents in POC settings, should be evaluated in remote communities with limited resources.

## Supplemental file

Supplemental materials
